# Soundboard-trained dogs produce non-accidental, non-random and non-imitative two-button combinations

**DOI:** 10.1038/s41598-024-79517-6

**Published:** 2024-12-09

**Authors:** Amalia P. M. Bastos, Zachary N. Houghton, Lucas Naranjo, Federico Rossano

**Affiliations:** 1 Department of Cognitive Science, University of California, San Diego, USA; 2 Department of Psychological & Brain Sciences, Johns Hopkins University, Baltimore, USA; 3 Department of Linguistics, University of California, Davis, USA; 4 Statistics and Operational Research Department, Universitat de València, Valencia, Spain; 5 CleverPet, Inc, San Diego, CA USA

**Keywords:** Interspecies communication, Augmentative interspecies communication (AIC), Soundboard, Dogs, Citizen science, Psychology, Animal behaviour

## Abstract

Early studies attempting interspecies communication with great apes trained to use sign language and Augmented Interspecies Communication (AIC) devices were limited by methodological and technological constraints, as well as restrictive sample sizes. Evidence for animals’ intentional production of symbols was met with considerable criticisms which could not be easily deflected with existing data. More recently, thousands of pet dogs have been trained with AIC devices comprising soundboards of buttons that can be pressed to produce prerecorded human words or phrases. However, the nature of pets’ button presses remains an open question: are presses deliberate, and potentially meaningful? Using a large dataset of button presses by family dogs and their owners, we investigate whether dogs’ button presses are (i) non-accidental, (ii) non-random, and (iii) not mere repetitions of their owners’ presses. Our analyses reveal that, at the population level, soundboard use by dogs cannot be explained by random pressing, and that certain two-button concept combinations appear more often than expected by chance at the population level. We also find that dogs’ presses are not perfectly predicted by their owners’, suggesting that dogs’ presses are not merely repetitions of human presses, therefore suggesting that dog soundboard use is deliberate.

**Supplementary Information:**

The online version contains supplementary material available at 10.1038/s41598-024-79517-6.

## Introduction

Scientific research on interspecies communication has a long and fraught history. Early studies attempted to teach language – both vocally^1,2^ and gesturally^3–5^ – to enculturated apes reared in human environments. These studies were heavily criticised for several reasons: methods were often inconsistent and underreported, caretakers and scientists alike tended to overinterpret animals’ behaviour, and rearing conditions in human environments is both dangerous and detrimental to apes (for an overview, see^1^). Anecdotes of interesting behaviours, such as chimpanzee Washoe’s signing of “water” and “bird” upon seeing a swan, led to claims of linguistic productivity in nonhuman animals^6^, which were quickly met by deflationary alternative explanations^7^. Even scientists who were themselves involved in this field of research ultimately argued that signing apes may have been simply imitating their caretakers’ hand signs, rather than demonstrating symbolic comprehension^8^. Following these criticisms, some researchers successfully implemented controlled laboratory experiments to shift the nature of this research from its purely anecdotal origins, and extended the field beyond just apes to also include parrots capable of vocal mimicry^9,10^.

Still other researchers turned to the use of augmentative interspecies communication (AIC) devices, such as lexigrams, magnetic chips, and buttons (for a review, see 6, 11). This method did not require vocal mimicry or fine manual motor skills, therefore extending its applicability to a wide range of species. Additionally, it allowed for greater separation between subjects and human trainers, and more rigorous training and data collection procedures^11^. These studies demonstrated that some animals – including apes^12,13^, dolphins^14^, and professionally-trained dogs Sofia^15^ and Laila^16^ – can learn to use AIC devices to make communicative requests, by associatively pairing labels with their effects in the world. For example, Sofia would reliably press individual voice-recorded keys for words such as “play” and “walk” to request their associated actions^15^, and both Sofia and Laila were sensitive to a human’s visual perspective when they used keys to communicate their requests^16^. However, they were not free of their own criticisms: this method was still susceptible to behavioural overinterpretation and the Clever Hans effect^17^, whereby humans unintentionally cue animals on what to do next, leading to the predicted behaviour by means other than the animal’s true comprehension of the task^11^.

Perhaps unbeknownst to the scientific debates surrounding interspecies communication, in recent years thousands of dog owners have begun training their pets with button soundboards^1^. Beyond making single button-presses for requests, however, owners report that their soundboard-trained pet dogs press buttons voicing labels for abstract concepts such as “more”, “later”, and “help”, and producing recurring sequences of two or more button presses, suggesting soundboard use that is closer to that observed in lexigram-trained apes^12^. Recent findings show that soundboard-trained dogs can recognize some of the word labels recorded onto their soundboards, responding appropriately when these are pressed either by their owner or by an unfamiliar person, in the absence of any additional contextual cues^18^. For example, soundboard-trained dogs were more likely to engage in playful behaviours upon having either their owner or an unfamiliar person press the button voicing the word “play”, compared to buttons voicing words unrelated to playing.

In order to determine whether this emerging global citizen science trend reflects a viable case of interspecies communication, we examine a dataset containing button presses made by dogs and their owners to determine the likely production mechanisms underlying dogs’ button presses. If pet dogs are using soundboards to communicate with their owners, then at the very least we expect that their presses should be: (i) non-accidental, suggesting that dogs’ pressing actions are deliberate and not the result of unrelated behaviours that might result in unintended pressing of particular soundboard buttons, (ii) non-random, suggesting that dogs do not indiscriminately press any buttons for rewards as a trained command, and (iii) non-identical to their owners’ presses, suggesting that dogs do not simply repeat their owners’ presses through social learning strategies such as stimulus enhancement or imitation. We test these predictions using a large dataset containing dog and owner soundboard presses.

## Methods

### Data collection

Owners of soundboard-using pets were asked to manually report button presses made by their dogs and by themselves using a purpose-built mobile application, either as they occurred, or from video they captured for annotation. Owners were instructed to report all presses, both by their dogs and by themselves, were provided with instructions on how to correctly report presses through the application, and were offered one-on-one support with researchers to address individual questions regarding data collection. No specific instructions were provided to owners on which button labels to provide to their animals, in line with our non-prescriptive approach to this research project^9^. Informed consent was obtained from owners participating in the study, and all methods were performed in accordance with the relevant guidelines and regulations. This study was considered exempt from IRB approval under 45 CFR 46.104(d) by the UCSD HRPP (protocol submission #805351).

Subjects had different levels of experience and different levels of engagement with their soundboards. Nevertheless, to ensure that the dataset included only legitimate data and dogs with reasonable experience of soundboard use, only subjects with 200 or more reported soundboard interactions were included in our analyses. This resulted in a dataset of 194,901 soundboard interactions (hereafter, “dog pressing events”) by 152 pet dogs, of which 56,676 (29.08%) were multi-button combinations, and a further 65,682 soundboard interactions performed by their owners (hereafter, “modelling events”), over the span of 21 months. Individual dogs’ presses were recorded for a median of 98 days, including gaps in activity with days where no presses were logged for either the dogs or their owners. For each dog, we recorded a median of 10.9 presses per logged day. Days with the most presses (upper 25% quartile) across all dogs contained from 17.8 presses in one day up to a maximum average of 90 presses per day. The labels recorded onto dogs’ soundboard buttons, and the layouts of their soundboards, were decided by their owners. Labels were sorted into 68 broad concept categories (e.g., “kibble”, “dinner”, and “food” labels were grouped under the “FOOD” concept category), which were pre-determined and embedded into the mobile application, such that owners themselves selected the appropriate category for each of their button labels. To distinguish between the labels voice-recorded onto the buttons (e.g., “kibble”) and the concept category these labels belong to (e.g., “FOOD”), we write out concept category names in capital letters.

### Social learning model

We examined the effect of owner modelling on the number of spontaneous dog button presses, to determine the extent to which dogs were simply repeating their owners’ presses of the same buttons, with no regard for the labels recorded onto the buttons. We used a Bayesian negative binomial model using brms^19^, implemented in R 4.4.1^20^. The model equation is given below:

Dog Presses ∼ Modelling Events ∗ Concept + (1 + Modelling Events ∗ Concept | Subject).

A negative binomial model was used because the outcome variable was count data and the variance was substantially higher than the mean (which is a violation of one of the assumptions of the Poisson distribution).

### Randomness index

We calculated a Randomness Index (RI) value for each dog given their multi-button presses on their soundboard, to determine the extent to which dogs were pressing buttons at random (see SI Methods for additional detail). RIs can determine the non-randomness of real-world networks based on the negative correlation between the Local Clustering Coefficient of a node and the degree of that same node^21^. For each soundboard combination network, we then generated 1,000 random simulated networks with the same number of nodes and links using the igraph^22^ package in R 4.4.1^20^. We compared the randomly generated network RIs with the RIs for their real counterparts, expecting that if dogs were pressing buttons in a non-random fashion, then the RI values for their real soundboard networks should be smaller than those for the average value extracted from 1,000 bootstrapped equivalent randomly-generated networks by a difference of at least 0.2. Comparisons were made using a paired Bayesian t-test using the BayesFactor package^23^ in R 4.4.1^20^.

### Two-button concept combination model

Importantly, non-random presses can still be produced accidentally by dogs moving across or onto their soundboards in some repetitive but non-deliberate manner. In order to determine whether dogs’ multi-button presses were generated non-randomly, we investigated the extent to which particular button concept combinations might appear repeatedly at the population level, considering only the 16 concepts that were provided most commonly across all dogs’ soundboards. Given that each dog’s soundboard was organised differently in terms of its button layout, and located in different parts of their respective homes and in different orientations, accidental stepping on buttons would predict a uniform distribution of two-button combination concepts at the population level. On the other hand, non-accidental pressing of meaningful buttons might lead to population-wide preferences for some button concepts over others, and potentially preferred two-button combinations at the population level.

To this end, we examined whether some combinations of button concepts occurred more frequently than others using a Bayesian negative binomial model using the brms package^19^ in R 4.4.1^20^. The model equation is given below:

Combination Frequency ∼ (1 + Combination ID + offset(rel prob) | Subject + (1|Combination ID)).

Note that we use a random-intercepts model because there are a large number of different combinations. In Bayesian models, partial pooling pulls random-effects closer to the average, such that groups with few data points are pulled towards the mean. In other words, for a coefficient estimate to be non-zero, there must be sufficient evidence to overcome the shrinkage. This is beneficial since we don’t want to draw strong conclusions based on only a few data points.

Given the size of our dataset, we subsetted the dataset by concept, excluding everything except the 16 concepts that were shared by the most dogs. Crucially, this subset was based not on how often dogs pressed each concept, but on whether the concept was present in the majority of the dogs’ soundboards (regardless of whether each dog pressed that concept frequently or not). In order to avoid conflating non-random combinations of presses with the individual buttons being pressed often, we calculated the relative probability of each individual button being pressed, and for each combination of buttons, we included the product of the relative probability of each button in the combination. The full output for this model is provided in the supplementary materials.

## Results

**Fig. 1:**
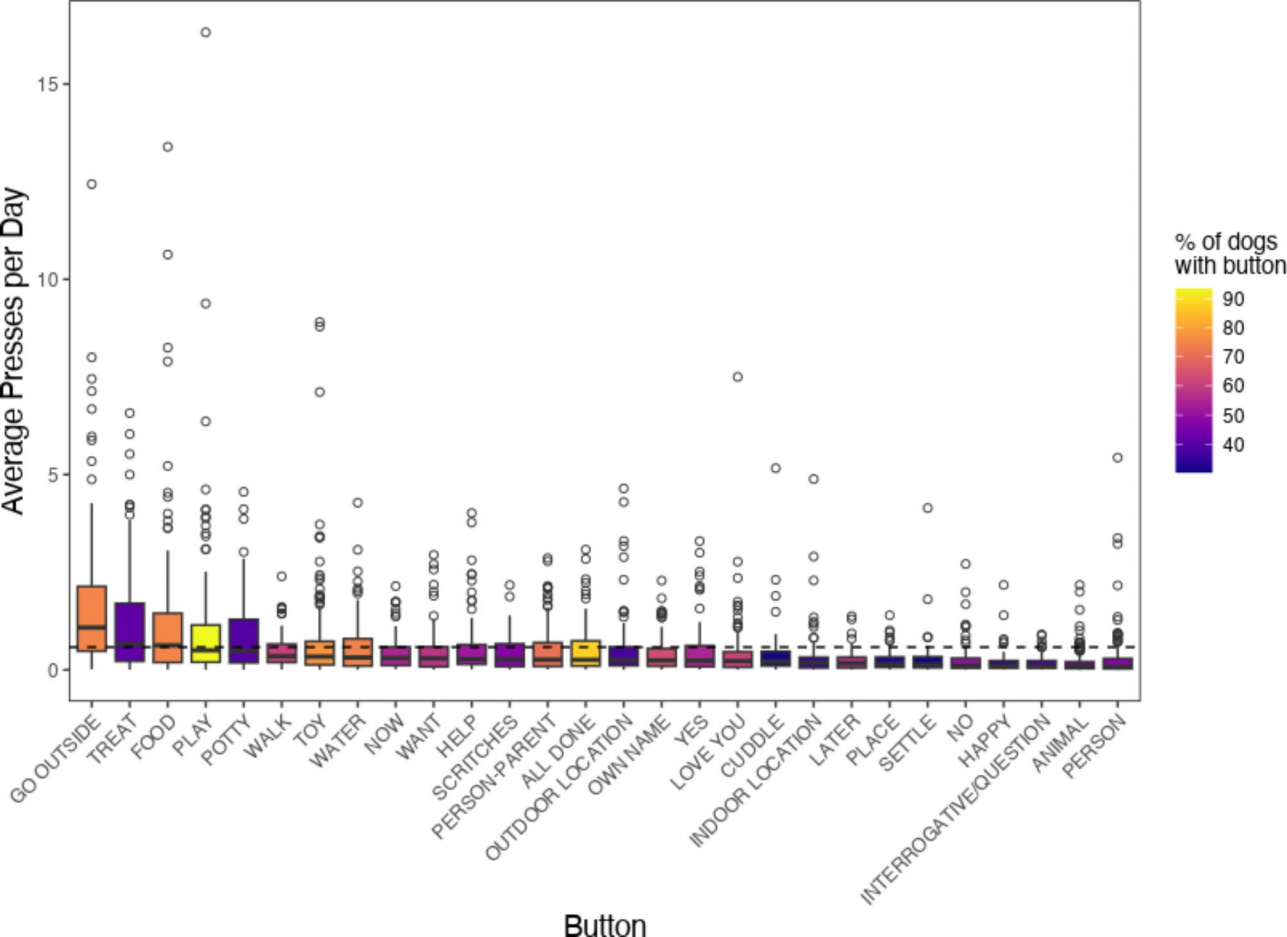
Average daily dog pressing events for all button concepts for all 152 pet dogs. Colours indicate the percentage of dogs whose soundboards contained said buttons.

The buttons most pressed by dogs were typically those for concepts relating to the animals’ routine activities and needs (Fig. 1). In order to determine whether dogs most commonly pressed the same buttons as their owners, we ran a mixed effects model examining the frequency of dog pressing events within each concept category, as determined by the owner’s modelling events, with individual slopes fitted for each subject. We found that there was only a minimal association between the identity of individual buttons pressed by dogs and the buttons pressed by their owners (β_modelling_ = 0.014, CI 2.5 = 0.011, CI 97.5 = 0.018).

**Fig. 2:**
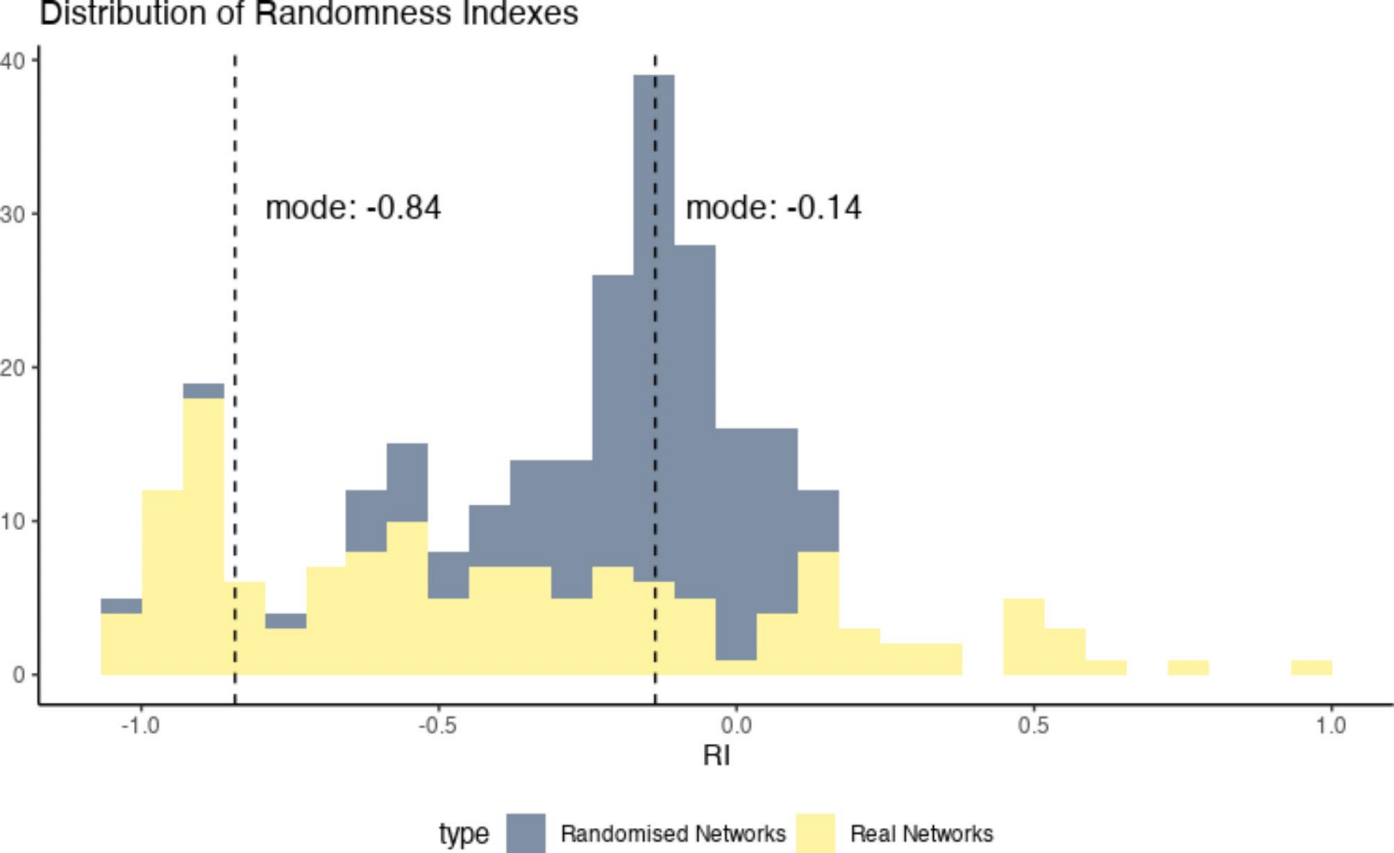
Randomness Index values calculated for real dogs’ soundboards (shown in yellow) and averaged from 1,000 randomly generated networks based on the number of nodes and link-probability for each real soundboard (shown in grey). Real networks’ RI values demonstrate some variability across subjects, although the mode RI value of -0.84 indicates non-randomness due to a strong negative correlation between node degree and Local Clustering Coefficient for most soundboard networks. On average, random networks have a RI centered around 0, while most real networks have a RI closer to -1. A RI closer to -1 suggests a structured network where central “hub” nodes are surrounded by many neighbouring nodes, which are not connected between themselves. Networks that are randomly generated do not show that hub-based structure.

To determine whether two-button sequences pressed by dogs were non-random, we first separated all sequences of three or more buttons into their constituent neighbouring pairs, for example splitting the three-button pressing event “want”+“food”+“outside” into two sequences, “want”+“food” and “food”+“outside”. The order of presses was disregarded, such that “food”+“outside” was equivalent to “outside”+“food”. This allowed us to generate an undirected and unweighted combination network for each subject’s soundboard, with button labels as nodes and two-button combinations generating the links between these nodes. We applied a Randomness Index (RI) measure^19^ to determine the extent to which dog’s two-button sequences were randomly produced. We found that the RIs calculated for dogs’ real combination networks were less random than those calculated for equivalent randomly generated bootstrapped networks for soundboards with the same number of nodes and link-probability (Bayesian paired t-test: BF = 72.73 ± < 0.01% error). As demonstrated in Fig. 2, although most dogs’ RI values deviated from that expected of random networks, this was not the case for every subject.

**Fig. 3:**
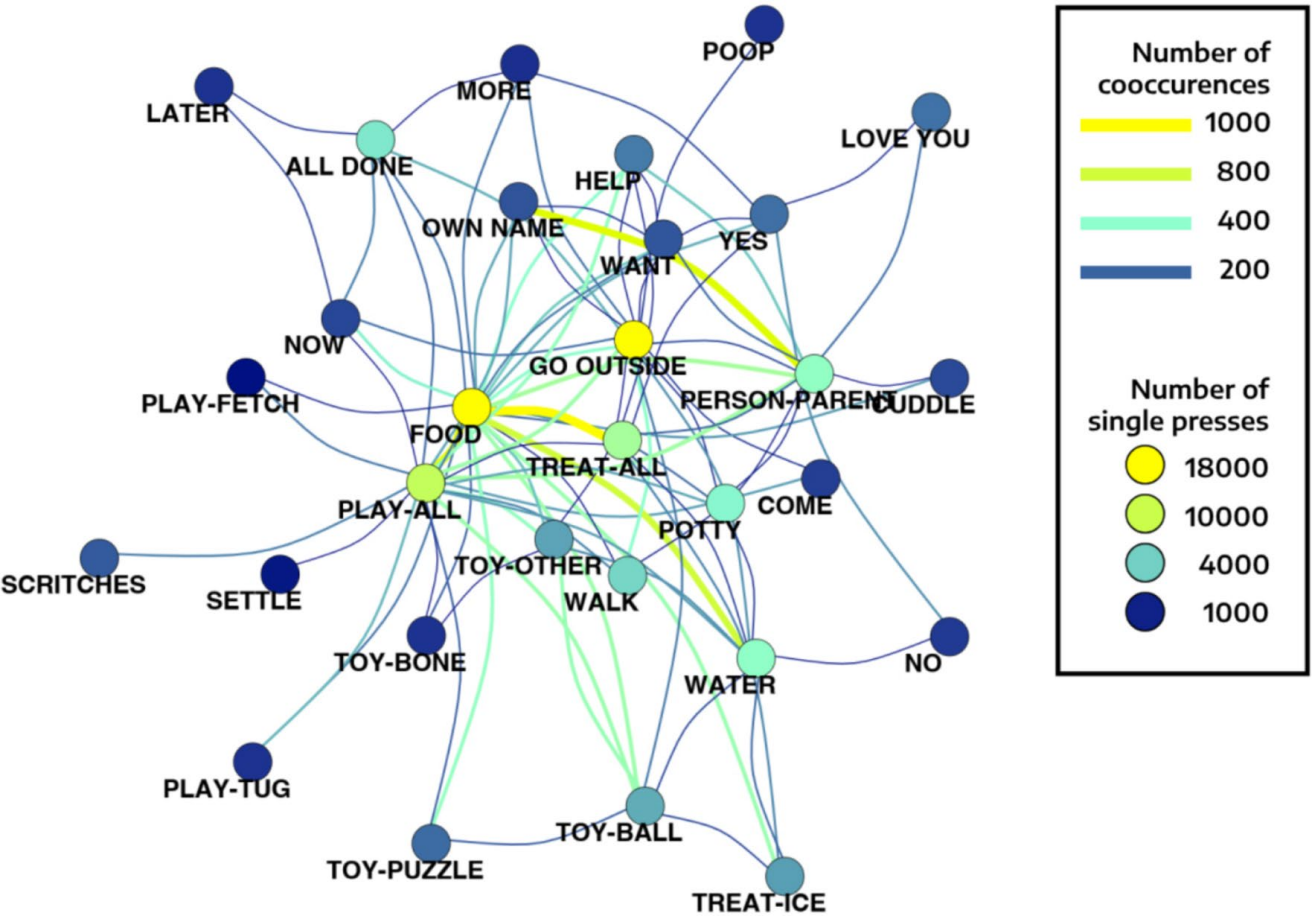
Population-level network for concept combinations that occurred a minimum of 100 times across dogs. More commonly observed combinations are represented by lines closer in warmer colours (range: 100 to 567; see figure key), as are the buttons pressed most often regardless of combinations (most common in yellow, least common in blue). Common concept combinations include “FOOD” + “TREAT” and “OWN NAME” + “WANT”. The latter was one of the most frequent combinations despite the fact that its constituent button concepts were among the least commonly pressed.

We ran a mixed effects model to investigate the frequency with which combinations of any two concepts appeared within two-button presses in the dataset, as determined by the concepts in that combination, fitting individual slopes for each subject. As before, we disregarded the order in which buttons were pressed within two-button sequences. We found that, even controlling for the relative probabilities of individual button concepts being pressed, button combinations were predicted by the concepts included within specific combinations. Therefore, dogs were more likely to produce some button concept combinations than others at the population level (Fig. 3). For example, the combination of concepts “FOOD” + “PLAY_ALL” occurred more often than expected by chance, even after controlling for its relative probability (β = 0.423, CI-2.5% = 0.106, CI-97.5% = 0.732). Other combinations that occurred more often than expected by chance were, for example “GO OUTSIDE” + “OTHER” (β = 0.398, CI-2.5% = 0.085, CI-97.5% = 0.705) and “HELP” + “OTHER” (β = 0.357, CI-2.5% = 0.077, CI-97.5% = 0.609). Examples of combinations that occurred less often than expected by chance were “LATER” + “LOVE YOU” (β = -0.442, CI-2.5% = -0.805, CI-97.5% = -0.100) and “NOW” + “WANT” (β = -0.461, CI-2.5% = -0.792, CI-97.5% = -0.135).

## Discussion

Our results show that owner-trained pet dogs can use soundboards to make non-random and deliberate button presses which are not simply repetitions of the presses made by their owners. Furthermore, at the population level, soundboard-trained dogs were more likely to produce two-button combinations for some concept pairs than others, despite individual subjects having soundboards with different layouts. This suggests that several soundboard-trained pet dogs successfully associate different outcomes to individual buttons, although the extent to which these outcomes match the meanings intended by their language-using owners is currently being investigated experimentally^9,18^.

The large number of presses reported in the dataset, and the much larger number of dog pressing events compared to modelling events, might suggest that presses were unlikely to be individually cued or prompted by owners. Additionally, if presses were performed as a trained command, then dogs should press buttons with no regard for their voiced labels or their position on the board, giving rise to random pressing across concepts at both the individual and population levels. This should give rise to random two-button combinations, with dogs pressing any two or more buttons in rapid succession to obtain the reward, again with no regard for the labels recorded onto them. Given that some concept pairs emerged more often than others at the population level across all dogs’ two-button combinations, this is unlikely to have been the case, even controlling for the fact that some buttons were pressed more than others. Even if dogs were more likely to make two-button combinations that led to a more positive emotional response by their owners (and hence were indirectly reinforced for doing so), it seems unlikely that all owners across all households would have preferentially responded to the same combinations, or even interpreted them in the same way.

Additionally, the frequency of dog button pressing per concept category was not meaningfully predicted by the owners’ presses for that same category, suggesting that dogs did not simply repeat their owners’ presses, and therefore did not simply attend to buttons preferred by their owners. However, we note that the dataset analysed for this study included only soundboard-experienced dogs, for whom over two hundred pressing events were reported. Although stimulus enhancement or other social learning heuristics are not the primary mechanism underlying these dogs’ presses, this may nevertheless be the case for dogs with little experience of soundboard devices, who are still learning the associations between button presses and their outcomes. Most owners reported training their dogs to use AIC soundboards by demonstrating the outcomes of different voice-recorded labels by producing a button press themselves and then performing its associated action, or “modelling”^9^. Whenever their dogs made any presses – whether deliberately or accidentally – they would then also perform the associated action. Anecdotally, owners reported reduced modelling over time once their dogs began pressing buttons unprompted. This training technique is different from that used with Sofia, who was first trained to press buttons as a trained command, which was always followed by their associated actions, and the command was extinguished once she began pressing spontaneously^15^. Ongoing work is investigating the extent of modelling required for dogs to press buttons voicing new labels, whether increased modelling improves dogs’ comprehension of these labels, and whether modelling does in fact decline over time as dogs gain button-pressing competence.

Although the present data demonstrates that dogs can produce non-random, non-accidental, and non-imitative button presses, questions remain as to subjects’ comprehension of the labels recorded onto said buttons. The issue of reference is beyond the scope of the present paper, but nevertheless a current topic of investigation with this population of dogs^9^. A demonstration that dogs do in fact consistently and correctly associate particular buttons with their relevant outcomes (e.g., that they expect their bowl to be filled after pressing “food”) is a critical prerequisite – but not a sufficient condition by itself – to determine whether soundboards can offer a viable means for interspecies communication. Thus far, a study has demonstrated that this population of soundboard-trained pet dogs can perform contextually appropriate behavioural responses to labels either recorded onto buttons or spoken out loud by their owner or an unfamiliar person^18^. Given the variability in network randomness found across subjects in the present study, future work should also investigate range in comprehension across dogs: we currently do not know whether learning to associate soundboard buttons with their respective outcomes is within the scope of any dog’s capacities, or whether, as is the case with gifted word-learning dogs that can learn verbal referents for hundreds or thousands of objects^24–26^, this is more limited to a subset of participants. Relatedly, future studies with even larger sample sizes will be better positioned to determine whether traits such as breed, age, or training background can be used to predict which dogs are most likely to engage with soundboard devices, or do so in a communicative manner.

Although owners were asked to report all button presses both by themselves and their dogs, it is still possible that presses may have been reported non-randomly: for example, owners could be more likely to launch the mobile application after observing button presses by their dog that they deemed “interesting”, over “uninteresting” ones. This could lead to biases in which concept buttons were most often reported at the level of individual dogs, but is unlikely to be the case at the population level, given that all dogs had different buttons and owners likely have different interpretations or perceptions of their dog’s presses. Our project is currently gathering continuous video and audio recordings of a subset of participants, which are annotated by trained researchers, to ensure a complete pressing record for a subset of participants^9^.

In sum, our results suggest that owner-trained dogs can press buttons on their soundboards in a non-accidental and non-random fashion, and that dogs do not simply repeat their owners’ presses. Therefore, our findings propose that dogs are differentiating between at least some of the buttons provided on their soundboards and, given the emergence of particular two-button concept combinations at the population level, that at least some dogs have associatively ascribed different meanings to different buttons. However, we note that our results do reflect considerable individual variation between subjects, with some soundboard networks approaching randomness, whilst others being extremely consistent in their two-button concept combinations. The observed patterns of use across a large number of dogs therefore propose that soundboard use by dogs is deliberate in nature, urging further investigation into this population of dogs and their pressing behaviours. In particular, future studies looking into the behaviours of dogs during soundboard use should investigate the extent to which dogs behave as if using buttons with communicative intent, for example, not barking simultaneously with their button presses (so they can be heard by their owners), socially referencing their owners by looking back at them following presses, and matching their body language to contextually-appropriate button presses (such as play bowing before or after pressing a button for “play” as opposed to a non-play-related button).

## Electronic supplementary material

Below is the link to the electronic supplementary material.

Supplementary Material 1

## Data Availability

Study pre-registration is deposited in the Open Science Framework, https://osf.io/kr7h9. Data and analysis scripts are available from Github, https://github.com/znhoughton/Modeling.
