# Supplementary material for: Soundboard-trained dogs produce non-accidental, non-random and non-imitative two-button combinations
Source: Sci Rep. 2024 Dec 9;14:28771. doi: 10.1038/s41598-024-79517-6 (PMC11628621; doi:10.1038/s41598-024-79517-6)
Supplement: Supplementary file 1 — Supplementary Material 1 [file 41598_2024_79517_MOESM1_ESM.pdf]

Supplementary Information

1) Social Learning Model

We examined the effect of modeling on the number of spontaneous button presses. We used a Bayesian negative binomial model using the brms package, implemented in R. The model equation is given below:

$$\text{Dog Presses} \sim \text{Modelling Events} * \text{Concept} + (1 + \text{Modelling Events} * \text{Concept} \mid \text{Subject})$$

A negative binomial model was used because the outcome variable is count data and the variance is substantially higher than the mean (which is a violation of one of the assumptions of the Poisson distribution).

There was minimal association between modeling events and button presses. While the results of our regression model are statistically significant (β_modeling=0.014, CI 2.5 = 0.011, CI 97.5 = 0.018), the number is so small that it is rather meaningless. The output of our regression model is included below in Table S1:

Parameter	Estimate	Est. Error	Q2.5	Q97.5
**Intercept**	**3.36**	**0.11**	**3.14**	**3.58**
**num_modeling_events**	**0.01**	**0.00**	**0.01**	**0.02**

Table S1. Negative binomial model of dog presses as a function of the number of times the buttons were modeled.

2) Randomness Index Analyses

The output for the Bayesian t-test comparing randomly generated networks and dogs' real soundboard networks is shown below:

```
Bayes factor analysis
--------------
[1] Alt., r=0.707 0<d<2    : 72.73± 0.01%
[2] Alt., r=0.707 !(0<d<2) : 0.006044676 ±0.01%

Against denominator:
  Null, mu = 0
```

For each subject, we had two R.I. values: real and random.

The real R.I. is made from observational data, while the random R.I. is made by simulating randomized interactions between the dog and the buttons.

Once we had both R.I.s for each dog, we computed the difference between the two values (random R.I. minus real R.I.).

R.I.s take values between -1 and 1, with 0 indicating randomness and -1 indicating non randomness, we expected the difference to be positive, which would suggest that real R.I.s are less random than random R.I.s.

For example, subject number 11642 has a real R.I. of -0.89 and a random R.I. of -0.04, resulting in a difference of 0.85.

3) Two-button Concept Combination Model

The full model results are given in Table S2 below.

	Estimate	Est. Error	CI-2.5%	CI-97.5%
**ALLDONE_FOOD**	0.028	0.153	-0.279	0.322
**ALLDONE_GOOUTSIDE**	0.156	0.148	-0.139	0.451
**ALLDONE_HELP**	-0.180	0.163	-0.508	0.135
**ALLDONE_LATER**	0.065	0.156	-0.248	0.361
**ALLDONE_LOVEYOU**	-0.107	0.151	-0.416	0.171
**ALLDONE_NOW**	0.200	0.146	-0.090	0.481
**ALLDONE_OTHER**	0.195	0.139	-0.076	0.460
**ALLDONE_OWNNAME**	-0.252	0.152	-0.549	0.045
**ALLDONE_PERSON_PARENT**	-0.080	0.121	-0.325	0.144
**ALLDONE_PLAY_ALL**	0.048	0.120	-0.190	0.282
**ALLDONE_WALK**	-0.190	0.131	-0.452	0.063
**ALLDONE_WANT**	-0.189	0.131	-0.446	0.064
**ALLDONE_WATER**	-0.295	0.152	-0.600	-0.003
**ALLDONE_YES**	-0.117	0.188	-0.494	0.244
**FOOD_GOOUTSIDE**	0.270	0.155	-0.029	0.568
**FOOD_HELP**	0.269	0.166	-0.065	0.586
**FOOD_LATER**	-0.174	0.157	-0.484	0.137
**FOOD_LOVEYOU**	0.007	0.158	-0.308	0.312
**FOOD_NOW**	0.035	0.180	-0.312	0.386
**FOOD_OTHER**	0.454	0.160	0.137	0.761
**FOOD_OWNNAME**	0.007	0.155	-0.301	0.308
**FOOD_PERSON_PARENT**	0.220	0.158	-0.089	0.522
**FOOD_PLAY_ALL**	0.423	0.160	0.106	0.732
**FOOD_WALK**	0.108	0.148	-0.192	0.388
**FOOD_WANT**	0.192	0.195	-0.197	0.558
**FOOD_WATER**	0.436	0.160	0.108	0.745
**FOOD_YES**	-0.070	0.189	-0.443	0.299
**GOOUTSIDE_HELP**	-0.035	0.164	-0.359	0.288

	Estimate	Est. Error	CI-2.5%	CI-97.5%
**GOOUTSIDE_LATER**	-0.086	0.131	-0.352	0.158
**GOOUTSIDE_LOVEYOU**	-0.195	0.178	-0.548	0.148
**GOOUTSIDE_NOW**	0.080	0.151	-0.226	0.382
**GOOUTSIDE_OTHER**	0.398	0.159	0.085	0.705
**GOOUTSIDE_OWNNAME**	0.015	0.150	-0.280	0.314
**GOOUTSIDE_PERSON_PARENT**	-0.003	0.151	-0.313	0.282
**GOOUTSIDE_PLAY_ALL**	0.290	0.142	-0.006	0.562
**GOOUTSIDE_WALK**	0.294	0.162	-0.025	0.601
**GOOUTSIDE_WANT**	0.036	0.151	-0.278	0.334
**GOOUTSIDE_WATER**	0.053	0.151	-0.243	0.351
**GOOUTSIDE_YES**	0.094	0.148	-0.202	0.378
**HELP_LATER**	-0.261	0.192	-0.641	0.103
**HELP_LOVEYOU**	-0.047	0.170	-0.402	0.278
**HELP_NOW**	-0.163	0.157	-0.471	0.151
**HELP_OTHER**	0.357	0.138	0.077	0.619
**HELP_OWNNAME**	-0.115	0.149	-0.415	0.169
**HELP_PERSON_PARENT**	0.093	0.160	-0.233	0.393
**HELP_PLAY_ALL**	0.286	0.148	-0.009	0.566
**HELP_WALK**	-0.062	0.199	-0.462	0.318
**HELP_WANT**	0.044	0.150	-0.259	0.337
**HELP_WATER**	-0.058	0.162	-0.368	0.252
**HELP_YES**	-0.237	0.151	-0.531	0.056
**LATER_LOVEYOU**	-0.442	0.178	-0.805	-0.100
**LATER_NOW**	-0.018	0.132	-0.288	0.238
**LATER_OTHER**	0.144	0.160	-0.181	0.449
**LATER_OWNNAME**	-0.326	0.199	-0.704	0.071
**LATER_PERSON_PARENT**	-0.436	0.148	-0.729	-0.142
**LATER_PLAY_ALL**	-0.067	0.158	-0.383	0.238
**LATER_WALK**	-0.201	0.181	-0.567	0.147
**LATER_WANT**	-0.439	0.229	-0.861	0.027
**LATER_WATER**	-0.198	0.162	-0.517	0.115
**LATER_YES**	-0.223	0.208	-0.641	0.179
**LOVEYOU_NOW**	-0.292	0.179	-0.658	0.062
**LOVEYOU_OTHER**	0.143	0.119	-0.098	0.375
**LOVEYOU_OWNNAME**	-0.049	0.141	-0.330	0.214
**LOVEYOU_PERSON_PARENT**	0.086	0.140	-0.197	0.358
**LOVEYOU_PLAY_ALL**	-0.051	0.121	-0.290	0.197
**LOVEYOU_WALK**	-0.197	0.176	-0.555	0.157
**LOVEYOU_WANT**	0.023	0.160	-0.294	0.340
**LOVEYOU_WATER**	-0.248	0.156	-0.570	0.047
**LOVEYOU_YES**	0.030	0.168	-0.298	0.354
**NOW_OTHER**	0.106	0.152	-0.198	0.401
**NOW_OWNNAME**	-0.437	0.154	-0.746	-0.141

	Estimate	Est. Error	CI-2.5%	CI-97.5%
**NOW_PERSON_PARENT**	-0.087	0.185	-0.474	0.273
**NOW_PLAY_ALL**	0.013	0.161	-0.300	0.324
**NOW_WALK**	-0.057	0.177	-0.410	0.276
**NOW_WANT**	-0.461	0.168	-0.792	-0.135
**NOW_WATER**	-0.180	0.160	-0.504	0.126
**NOW_YES**	-0.075	0.188	-0.440	0.292
**OTHER_OWNNAME**	0.205	0.146	-0.088	0.493
**OTHER_PERSON_PARENT**	0.270	0.139	-0.017	0.535
**OTHER_PLAY_ALL**	0.377	0.140	0.102	0.643
**OTHER_WALK**	0.057	0.139	-0.228	0.318
**OTHER_WANT**	0.332	0.126	0.075	0.570
**OTHER_WATER**	0.138	0.146	-0.150	0.418
**OTHER_YES**	0.099	0.151	-0.200	0.395
**OWNNAME_PERSON_PARENT**	0.557	0.137	0.284	0.822
**OWNNAME_PLAY_ALL**	0.205	0.138	-0.081	0.463
**OWNNAME_WALK**	-0.204	0.174	-0.546	0.133
**OWNNAME_WANT**	0.031	0.138	-0.248	0.296
**OWNNAME_WATER**	-0.187	0.167	-0.524	0.138
**OWNNAME_YES**	-0.156	0.167	-0.494	0.157
**PERSON_PARENT_PLAY_ALL**	0.254	0.124	0.006	0.490
**PERSON_PARENT_WALK**	0.038	0.144	-0.242	0.308
**PERSON_PARENT_WANT**	0.046	0.156	-0.273	0.348
**PERSON_PARENT_WATER**	-0.135	0.150	-0.432	0.154
**PERSON_PARENT_YES**	-0.030	0.145	-0.329	0.250
**PLAY_ALL_WALK**	0.064	0.145	-0.232	0.341
**PLAY_ALL_WANT**	-0.057	0.148	-0.353	0.227
**PLAY_ALL_WATER**	0.025	0.139	-0.247	0.288
**PLAY_ALL_YES**	-0.106	0.145	-0.394	0.169
**WALK_WANT**	-0.084	0.192	-0.481	0.277
**WALK_WATER**	-0.027	0.139	-0.309	0.230
**WALK_YES**	0.050	0.220	-0.384	0.466
**WANT_WATER**	-0.168	0.158	-0.489	0.136
**WANT_YES**	0.124	0.167	-0.210	0.448
**WATER_YES**	0.029	0.263	-0.490	0.547

Table S2. All possible two-button combinations involving the sixteen most commonly provided concepts on dogs' soundboards. Positive estimate values indicate more likely combinations, while negative estimate values indicate less likely combinations. Confidence intervals not overlapping zero suggest differences between estimated likelihoods and chance.
